# Post-operative infection and sepsis in humans is associated with deficient gene expression of γ_c _cytokines and their apoptosis mediators

**DOI:** 10.1186/cc10293

**Published:** 2011-06-28

**Authors:** Mary White, Vivienne Mahon, Robert Grealy, Derek G Doherty, Patrick Stordeur, Dermot P Kelleher, Ross McManus, Thomas Ryan

**Affiliations:** 1 Dept of Anaesthesia and Intensive Care Medicine, St James Hospital, James Street, Dublin 08, Ireland; 2 Dept of Clinical Medicine, Institute of Molecular Medicine, Trinity College, Dublin 02, Ireland; 3 Dept of Molecular Microbiology, Institute of Molecular Medicine, Trinity College, Dublin 02, Ireland; 4 Dept of Immunology, Institute of Molecular Medicine, Trinity College, Dublin 02, Ireland; 5 Service d'Immunobiologie-Hémobiologie-Transfusion, Hôpital Erasme, Université Libre de Bruxelles, Belgium

## Abstract

**Introduction:**

Lymphocyte homeostasis is dependent on the γ_c _cytokines. We hypothesised that sepsis in humans is associated with differential gene expression of the γ_c _cytokines and their associated apoptosis mediators.

**Methods:**

The study population consisted of a total of 60 patients with severe sepsis, 15 with gram negative bacteraemia, 10 healthy controls and 60 patients undergoing elective lung resection surgery. Pneumonia was diagnosed by CDC NNIC criteria. Gene expression in peripheral blood leukocytes (PBLs) of interleukin (IL)-2, 7, 15 and interferon (IFN)-γ, Bax, Bim, Bcl-2 was determined by qRT-PCR and IL-2 and IL-7 serum protein levels by ELISA. Gene expression of IL-2, 7 and IFN-γ was measured in peripheral blood leukocytes (PBL), cultured in the presence of lipopolysacharide (LPS) and CD3 binding antibody (CD3ab)

**Results:**

IL-2 gene expression was lower in the bacteraemia group compared with controls, and lower still in the sepsis group (*P *< 0.0001). IL-7 gene expression was similar in controls and bacteraemia, but lower in sepsis (*P *< 0.0001). IL-15 gene expression was similar in the three groups. Bcl-2 gene expression was less (*P *< 0.0001) and Bim gene expression was greater (*P *= 0.0003) in severe sepsis compared to bacteraemic and healthy controls. Bax gene expression was similar in the three groups.

In lung resection surgery patients, post-operative pneumonia was associated with a perioperative decrease in IL-2 mRNA (*P *< 0.0001) and IL-7 mRNA (*P *= 0.003). IL-2 protein levels were reduced in sepsis and bacteraemia compared to controls (*P *= 0.02) but similar in pneumonia and non-pneumonia groups. IL-7 protein levels were similar in all groups.

In cultured PBLs, IFN-γ gene expression was decreased in response to LPS and increased in response to CD3ab with sepsis: IL-7 gene expression increased in response to LPS in controls and to CD3ab with sepsis; Bcl-2 gene expression decreased in response to combined CD3ab and IL-2 with sepsis.

**Conclusions:**

Patients with infection and sepsis have deficient IL-2 and IL-7 gene expression in PBLs. Aberrant cytokine gene expression may precede the onset of infection.

## Introduction

The gamma chain (γc) family of interleukins (IL) that includes IL-2, IL-7, IL-15 and IL-21, regulate lymphocyte homeostasis. These cytokines act on distinct lymphocyte populations; IL-2 is a T cell growth factor, contributes to the development of regulatory T (T reg) cells [1]; IL-7 is critical for T and B cell development and is a potent lymphocyte survival factor [2]; IL-15 is a trophic factor for NK cells and CD8^+ ^T cell homeostasis [3]. The cellular effects of IL-2 and IL-7 are mediated by regulating Bcl-2, Bax and Bim mediated apoptosis [4,5].

This group has previously shown a link between effector cytokine gene expression in peripheral blood leukocytes (PBL) and human response to infection and patient response to surgery [6-10]. In these studies cytokine gene expression was assayed by reverse transcriptase polymerase chain reaction according to an established protocol [11]. This approach provides a unique *in vivo *insight to PBL function in humans that may not be reflected by cytokine protein levels in peripheral blood, as these proteins may emanate from a diverse range of other cells. However, these cytokine gene expression patterns did not include cytokines which regulate T cell homeostasis, and consequently did not account for the profound lymphocyte apoptosis reported in animal models of sepsis and in humans [12-14]. It is plausible that cytokines involved in regulating T cell homeostasis are involved in the immune deficiency linked with sepsis. Furthermore, the onset and evolution or resolution of sepsis may be related to dysregulation of these cytokines. We hypothesised the gamma c cytokine mediated response may be important in both the predisposition and occurrence of infection and sepsis in humans. Our primary endpoint was to determine whether sepsis in humans was associated with differential gene expression of gamma c family of cytokines. A secondary endpoint was to determine whether any differential gene expression of the gamma c cytokines preceded the clinical onset of infection.

We report gene expression of the γ_c _cytokines and associated regulators of apoptosis in PBLs of patients with sepsis, and gene expression in lung resection surgery patients. We also investigated the effect of exogenous IL-2 on cytokine gene expression in cultured mononuclear cells to determine whether IL-2 might plausibly be considered as an immune adjuvant for patient with sepsis. While we could link changes in cytokine gene expression to the occurrence of sepsis in response to infection, we were not clear whether this was an epiphenomenom, or was causal in nature. To clarify this issue we needed to study a group of patients who were likely to develop infection and study cytokine gene expression prior to the onset of infection. Thus, we chose thoracic surgery patients as a group with relatively frequent occurrence of post-operative pneumonia [15], in which we could draw blood samples before and after surgery, but prior to the onset of infection. Our results indicate that the occurrence of infection and sepsis are linked with deficient γ_c _family cytokines gene expression, specifically IL-2 and IL-7.

## Materials and methods

### Patient groups

#### Established infection (sepsis, bacteraemia)

We studied 60 consecutive patients presenting with severe sepsis or septic shock attending St. James's Hospital, Dublin. All patients met the criteria as defined by the American College of Chest Physicians/Society of Critical Care Conference [16]. Ethical approval for this study was obtained from the Ethics Committee of St. James's Hospital and informed written consent was obtained from each patient or relative. Exclusion criteria included the following: (1) pre-existing overt organ failure, (2) infection with human immunodeficiency virus, (3) patients with neutropenia as a result of chemotherapy, (4) patients receiving long-term treatment with steroids, (5) patients with trauma and burns, and (6) patients with non-Irish Caucasian ethnic background.

Severity of illness was characterized upon admission to ICU using the Simplified Acute Physiology Score (SAPS II) [17] and the Sequential Organ Failure Assessment (SOFA) [18] scoring systems and again on Day 7. Clinical and laboratory variables were collected on Day 1 and again on Day 7 in survivors. The source of infection precipitating ICU admission and the pathogenic organisms were documented. ICU death or survival to ICU discharge was recorded.

Fifteen consecutive patients, admitted to hospital with a documented Gram-negative bacteraemia on blood culture were enrolled if no organ failure or impending septic crisis was identified. Ten healthy staff members were identified as a control group.

#### Prospective infection cohort

A second study population consisting of 60 patients who underwent thoracotomy for lung resection surgery for non-infectious disease and without signs of acute respiratory infection were enrolled. Patients with pre-existing immunosuppression and those treated with anti microbials in the week preceding surgery were excluded. Postoperative patient care was provided by the primary care service in accordance with the established patient care pathways in our institution and without reference to the investigators. The diagnosis of post-operative pneumonia (POP) in the first week after surgery was based on the Center for Disease Control and Prevention definition [19].

#### Blood sampling

EDTA anticoagulated blood samples were taken within the first 24 h of ICU admission and again seven days later in the ICU patients. In bacteraemic patients, blood sampling was carried out within 24 h of the positive blood culture being reported. Blood samples were collected from controls at one time point. In the thoracotomy group, whole blood sampling was collected preoperatively (Day 0) and again 24 h (Day 1) and five days (Day 5) postoperatively. PBLs were prepared by density gradient centrifugation using Lymphoprep (Nycomed Pharma, Oslo, Norway). For RNA extraction, PBLs were lysed with RLT lysis buffer (Qiagen, West Sussex, England) and stored at -80^°^C. For flow cytometry, PBLs were used immediately. Serum was obtained from whole blood clotted for 30 minutes, and stored at -80^°^C until analysis.

Total RNA was isolated from lysed PBLs using a commercially available kit (Qiagen) following the manufacturer's instructions. To avoid amplification of contaminating genomic DNA, all samples were treated with RNase-free DNase (Qiagen) for 15 minutes. The quantity and purity of extracted RNA was measured by spectrophotometry (Eppendorf BioPhotometer, Hamburg, Germany).

Total RNA was reverse transcribed as previously described [11]. The PCR reactions were carried out in an ABI Prism 7000 (Applied Biosytems, Foster City, CA, USA) and all reactions were performed in triplicate as previously described [11]. Primers and probes used in this study were synthesized at Applied Biosystems. β-Actin, IL-2, IL-7, IL-15, IL-21, Bcl-2, Bim, Bax primers and probes were designed and customised as per Stordeur *et al*. [11]. The DNA standards for β-Actin, IL-2, IL-7, IL-15, IL-21, Bim, Bax, Bcl-2 consisted of a cloned PCR product that included the quantified amplicon prepared by PCR from a cDNA population containing the target mRNA as per Stordeur *et al*. [11]. To quantify transcript levels a standard curve was constructed, for each PCR run, for each selected mRNA target from serial dilutions of the relevant standard as previously described [11]. Results were then expressed in absolute copy numbers after normalisation against β-Actin mRNA (mRNA copy numbers of cytokine mRNA per 10 million β-Actin mRNA copy numbers). Normalisation calculation = (Target Gene Copy Number/β-Actin Copy Number)*7.

#### Cytokine serum protein measurement by Enzyme Linked Immunosorbant Assay (ELISA)

Serum was collected from patients and controls at the same timepoints as blood was collected for gene expression. Serum IL-2 and IL-7 concentrations were measured by in triplicate by ELISA (R&D Systems, Minneapolis, MN, USA). The lower limit of detection of IL-2 was 11.2 pg/mL and IL-7 was 0.50 pg/mL.

#### *In vitro *stimulation of peripheral blood leukocytes

Blood samples were collected from five healthy controls and five patients with severe sepsis. PBLs were isolated and cultured in 24-well plates at 10^6 ^cells/ml in RPMI 1640 supplemented with 10% (vol/vol) FCS and 2 mM glutamine 4-(2-hydroxyethyl)-1-piperazineethanesulfonic acid (HEPES). Cells were incubated in medium alone or stimulated with either 1 μg/ml lipopolysaccharide (LPS) (Control LPS and Sepsis LPS) for 24 h (to activate monocytes and B cells), 3 μg/ml anti-CD3 monoclonal antibody (αCD3ab) (Control CD3 and Sepsis CD3) for 4 h (to activate T cells), or 10 ng/ml phorbol myristate acetate + 1 μM ionomycin (PMA+iono) (Control PMA and Sepsis PMA) for 4 h (to activate all cells). Each experiment was performed in the absence and presence of 50 U recombinant IL-2 (rIL-2). 2 × 10^5 ^PBLs were harvested immediately for flow cytometry and remaining cells were lysed. Total RNA was isolated; reverse transcribed as previously described [11] and mRNA assayed for IFN-γ, IL-2, IL-7 and Bcl-2.

#### Flow cytometry

Flurochrome-labelled monoclonal antibodies specific for CD3, CD4, CD8, CD56, CD127, CD25, CD19, IFN-γ and IL-2 and isotype-matched control antibodies were obtained from BD Biosciences. PBLs (2 × 10^5^) were stained for cell surface antigen expression at 4^°^C in the dark for 30 minutes, then washed twice in 2 mL phosphate-buffered saline containing 1% bovine serum albumin and 0.01% sodium azide (FACS Wash) and subsequently fixed in 200 μL of 1% paraformaldehyde (Sigma-Aldrich, Arklow, Ireland). Isotype-matched fluorescently-labelled control antibodies were used to determine background levels of staining. Lymphocytes and monocytes were identified by characteristic forward scatter and side scatter parameters and T cells, NK cells and monocytes were defined as CD3^+^, CD3^-^CD56^+ ^and CD14^+ ^populations, respectively. Results are expressed as % positive of gated population.

#### Statistical analysis

The Wilcoxon rank sum test was used to analyse the differences between groups for continuous variables. Categorical variables were analysed by Chi Square test and Fishers exact test as appropriate. Results from cell culture experiments were analysed with a fully factorial MANOVA, with post hoc contrast to identify differences between control and septic patients, where multivariate analysis identifying a difference between these groups. Data analysis was performed using the JMP statistical package (SAS, Cary, NC, USA).

## Results

**Figure 1:**
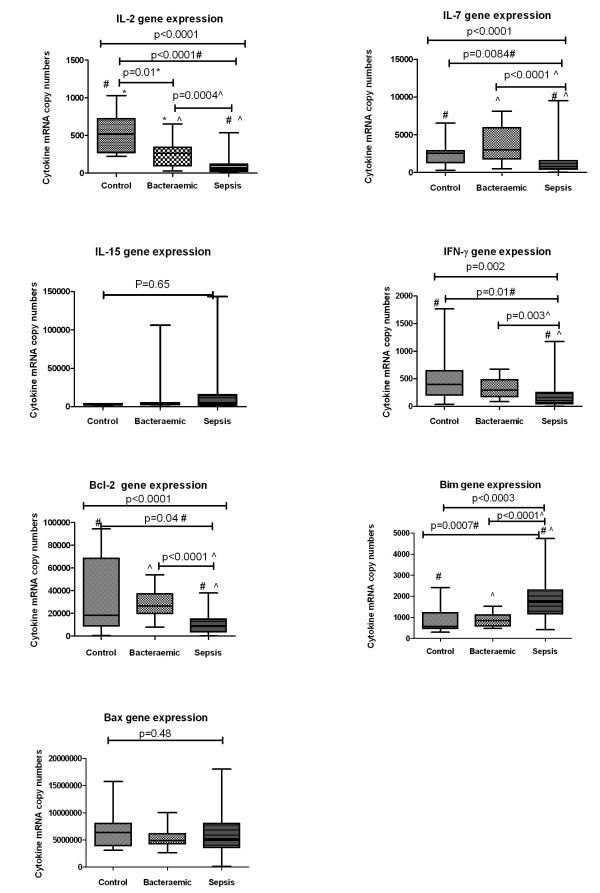
**Cytokine gene expression levels in sepsis (*n *= 60), bacteraemia (*n *= 15) and controls (*n *= 10) as measured with qRT-PCR**. IL-2, IL-17, IL-15, IFN-γ, Bcl-2, Bim and Bax are expressed as copy numbers of cytokine mRNA normalised to per 10 million β-Actin mRNA copy numbers. Values represented as medians and centiles 10 to 90% in parenthesis. Between groups analysis is by Wilcoxon Rank Sum test. Pairwise comparisons between control and bacteraemic (*), control and sepsis (#), p values are indicated where positive.

Demographic variables, site of infection and severity of illness scores from 60 septic, 15 bacteraemic and 10 healthy controls are given in Table 1. IL-2 gene expression was lower in bacteraemia patients compared with controls, and lower still in patients with sepsis (Figure 1); IL-7 gene expression was similar in control and bacteraemic patients, but lower in patients with sepsis; IL-15 gene expression was similar in the three groups. IL-21 mRNA was not detected in PBLs of any study groups. There was no relation between gender, age and IL-2 and IL-7 mRNA levels.

**Table 1:** Patient demographics of the three study groups, sepsis (*n *= 60), bacteraemia (*n *= 15) and healthy controls (*n *= 10)

	Sepsis	Bacteraemia	Control	*P*
**N**	60	15	10	
**Age**	54 (72 to 80)	73 (70 to 82)		ns
**Gender (Female)**	27 (48%)	9 (60%)		ns
**Apache**	25 (21 to 28)	7 (6 to 8)		0.0001
**SOFA**	10 (12 to 14)	1 (1 to 2)		0.0001
**Septic Shock**	58 (98%)	0		0.0001
**Mortality**	18 (30%)	0		0.002
**Respiratory Infection**	25 (42%)	2 (13%)		
**Abdominal Infection**	28 (48%)	4 (27%)		
**Urinary**	0	6 (40%)		
**Other Infection**	6 (10%)	3 (20%)		0.0001

Patient demographics of the three study groups, Sepsis (*n *= 60), Bacteraemia (*n *= 15) and Healthy Controls (*n *= 10). N denotes number in each group. All continuous values are median and inter-quartile range. Categories are stated as absolute numbers with percentages in parentheses. Apache, Acute Physiology and Chronic Health Evaluation; SOFA, Sequential Organ Failure Assessment.

Bcl-2 gene expression was greatest in bacteraemic patients, intermediate in controls and the least in septic patients; Bim gene expression was increased in sepsis compared to bacteraemia and controls; Bax gene expression was similar in all three groups (Figure 1).

There was no relation between severity of organ failure and/or outcome in patients with sepsis and IL-2, IL-7, IL-15, Bim, Bax or Bcl-2 gene expression.

**Figure 2:**
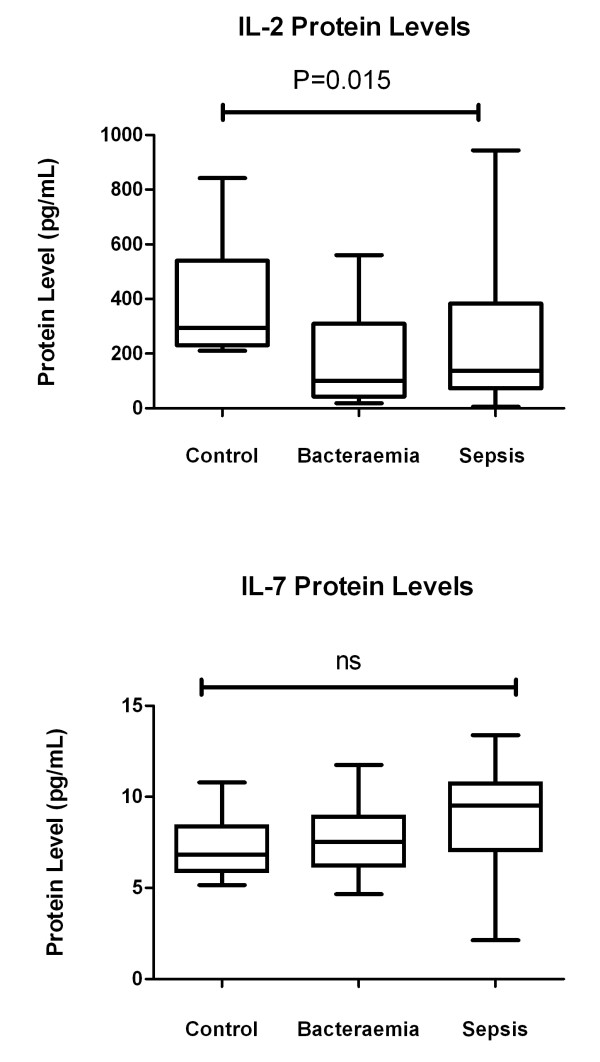
**Cytokine protein levels in patients with sepsis (*n *= 56), bacteraemic (*n *= 15) and control (*n *= 10) study groups**. Interleukin-2 (IL-2) and Interleukin-7 (IL-7) are expressed as picograms per milliliter (pg/mL). * denotes *P-*value < 0.05. N denotes number of subjects in each group. All values are median and interquartile range in parenthesis. Between group comparisons is by Wilcoxon Rank Sum test.

IL-2 protein levels were greater in healthy controls compared to patients with established infection (Figure 2). IL-7 protein levels were similar in the three groups. There was no correlation between cytokine mRNA levels and respective cytokine protein levels. There was no relation between survival or severity of organ failure and cytokine protein levels.

**Figure 3:**
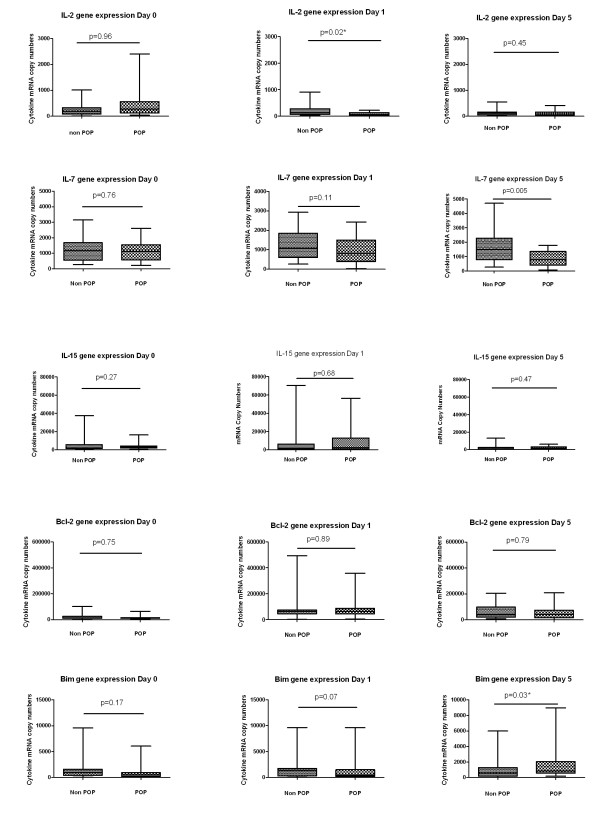
**Cytokine mRNA gene expression in thoracic surgery patients**. Cytokine mRNA gene expression was analysed at baseline Day 0 (24 hours pre surgery), Day 1 (24 hours post surgery) and Day 5 after surgery. POP, post-operative pneumonia (*N *= 19). Non POP, non post-operative pneumonia (*N *= 41). IL-2, IL-7, IL-15, Bcl-2 and Bim are expressed as copy numbers of cytokine mRNA per 10 million β-Actin mRNA copy numbers. Values represented as medians and centiles 10 to 90% in parenthesis. Between groups analysis is by Wilcoxon Rank Sum test. N denotes number of patients in each group.

Demographic variables, functional impairment scores and information on the type of surgery performed for 60 elective thoracic surgery patients are given in Table 2. In thoracic surgery patients, POP was associated with reduced IL-2 gene expression on the first day after surgery and lower IL-7 gene expression on the fifth day after surgery (Figure 3). When the relative changes in cytokine mRNA were considered, there was a significant link between a decrease in IL-2 and IL-7 gene expression on the first day after surgery and the subsequent occurrence of POP (Table 3). Allowing for the multiple comparisons of cytokine gene transcription, at three time points, the perioperative change in IL-2 would still retain statistical significance while the significance of change in IL-7 would be borderline.

**Table 2:** Patient demographic data in lung resection surgery study group

		Non POP	POP	*P*
**Gender, M/F**		29/12	13/6	0.86
**Age in years**		64.5 +/- 1.34	63.7 +/- 1.9	0.74
**Body Mass Index, kg/m^2^**		25.9 +/- 0.92	27.65 +/- 1.35	0.29
**Karnofsky index score, mean +/-SD**		90.24 +/- 1.17	88.95 +/- 1.72	0.54
**NSCLC, *n *(%)**		24 (58%)	8 (47%)	0.82
**Currently smoking**		16 (39%)	8 (42%)	
**Recent Cessation**		15 (36%)	8 (42%)	
**Non Smoker**		10 (24%)	3 (16%)	0.18
**Cardiovascular co morbidity, *n *(%)**		14 (34%)	7(37%)	0.57
**Preoperative FEV_1_, (%)**		82.12 +/- 2.28	82.34 +/- 3.36	0.95
**Type of operation**	Lobectomy	39	14	0.99
	Pneumonectomy	2	5	0.03*
**Neoadjuvant chemotherapy**		5 (12.2%)	2 (10.5%)	0.96
**Perioperative anti-microbial cover**		41(100%)	19 (100%)	ns
**Anaesthesia time in minutes**		233.0 +/- 40	238.6 +/- 52	0.51
**One lung Ventilation time in minutes**		85.5 +/- 14.9	88.0 +/- 9.6	0.19
**Post-operative PPV**		2	1	ns

Patient demographic data in lung resection surgery study group. Absence of post-operative pneumonia (non-POP); post-operative pneumonia (POP). Post-operative positive pressure ventilation (Post-operative PPV). NSCLC, Non small cell lung cancer. Categories are stated as absolute numbers with percentages in parentheses. Values given as mean +/- SD or number (%) unless otherwise indicated. FEV_1_, forced expiratory volume in one second.

**Table 3:** Relative Change in cytokine mRNA in the first 24 hours after surgery

	POP	Non POP	*P*
**IL-2**	6.7 (2.1 to 28.1)	1.1 (0.3 to 4.7)	< 0.0001
**N**	**14**	**37**	
**IL-7**	1.3 (0.9 to 6.2)	0.9 (0.5 to 2.1)	0.003
**N**	**18**	**41**	

Relative change in cytokine mRNA gene expression in first 24 hours after surgery. These data represent the ratio of pre-operative mRNA to post-operative mRNA; the preoperative value divided by the post-operative value. All values are quotes as median and 10^th ^to 90^th ^centile range. POP, post-operative pneumonia, Non POP, uneventful recovery; mRNA, messenger ribonucleic acid. Comparison is by Mann Whitney. N denotes the number of patients in each group.

Pre-operative IL-2 and IL-7 mRNA values of patients in the thoracic surgery study were compared with patients in the sepsis study. Median Pre-operative IL-2 mRNA copy numbers were similar to those of bacteraemic patients, less than healthy controls, and greater than patients with sepsis (Wilcoxon rank sum test *P *< 0.0001). Median Pre-operative IL-7 mRNA copy numbers were similar to those of bacteraemic patients and healthy controls, and greater than patients with sepsis (Wilcoxon rank sum test *P *< 0.0001). Median Pre-operative Bcl-2 mRNA copy numbers were similar to those of bacteraemic patients and healthy controls, and greater than patients with sepsis (Wilcoxon rank sum test *P *< 0.0001). Bax mRNA levels were similar in all four groups.

Serum IL-2 and IL-7 protein levels were similar in POP and non POP groups both before surgery and Day 1 and Day 5 after surgery.

Cell surface expression of the IL-2 receptor in T cells CD4^+^CD25^+^was similar in sepsis and control samples (Table 4). The IL-7 receptor, CD127^+^, expression is increased on CD3^+^lymphocytes from patients with sepsis but reduced on NK cells compared to control samples (Table 4).

**Table 4:** Cell surface antigen staining % total peripheral blood leukocytes

		Control	Sepsis	*P*
**N**		**5**	**5**	
**Monocytes**		4.9 (2.5 to 6.9)	5.2 (2.7 to 7.1)	**0.9**
**Lymphocytes**				
	iNKT	0.8 (0.2 to 1.0)	0.2 (0.1 to 0.7)	0.05*
	B Cells	6.1(5.2 to 12.8)	16.2 (1.9 to 18.1)	0.62
	NK cells	2.0 (0.1 to 8.0)	4.6 (0.6 to 12.7)	0.25
	CD56+CD127+	66.5 (62.7 to 73.9)	20.9 (3.9 to 53.9)	0.01*
	CD56^+^	1.6 (0.3 to 3.4)	0.6 (0.3 to 3.8)	0.40
	CD3+	83.9 (77.5 to 92.7)	61.3 (54.0 to 67.2)	0.009*
	CD3+CD4+	53.65 (25.7 to 66.4)	77.7 (65.5 to 84)	0.04*
	CD3+CD4+CD25+	19.85 (3.4 to 25.2)	11.4 (1.8 to 16.5)	0.25
	CD3+CD8+	30.7 (19.5 to 45.9)	16.1 (14.4 to 33.6)	0.14
	CD3+CD127+	80.5 (49.3 to 89.3)	97.2 (87.2 to 98.6)	0.01*

Cell surface antigen staining % total peripheral blood leukocytes. All values are quoted as percentage values of total peripheral blood leukocytes. All lymphocyte subsets are quoted as total gated lymphocytes. All values are quoted as median and 10^th ^to 90^th ^centile range. CD4+CD25+, CD4+, CD8+ are quoted as percentages of T Lymphocytes. NK, natural killer cells, iNKT cells; Between group comparison is by Mann Whitney.

**Figure 4:**
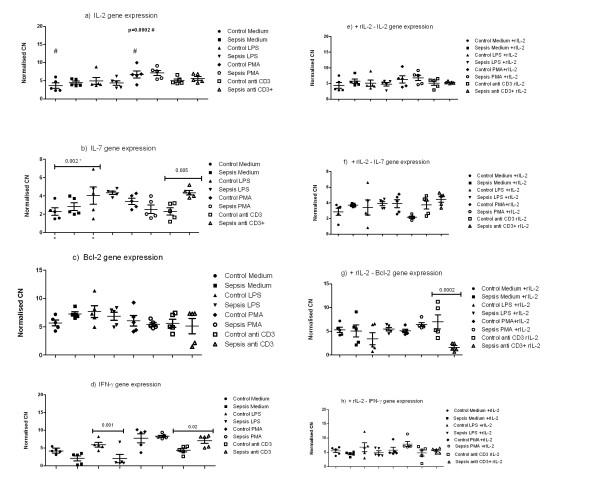
**Cytokine mRNA gene expression after *in vitro *stimulation of PBLs from septic (*n *= 5) and control (*n *= 5) patients**. Interleukin (IL) -2, 7, Bcl-2 and Interferon gamma (IFN-_γ_) gene expression are expressed as normalised copy numbers of cytokine mRNA per 10 million β-Actin mRNA copy numbers is plotted on y axis. PBL's were incubated with medium alone, or stimulated with 1 μg/ml lipopolysaccharide (LPS), or stimulated with 3 μg/ml anti-CD3 monoclonal antibody (CD3) or alternatively stimulated with 1 μg/M PMA and ionomycin. Type of stimulation is plotted on × axis. Figure 1 a, b, c, d *in vitro *stimulation of PBLs from control and septic patients in absence of recombinant IL-2 (rIL-2) and Figure 1e, f, g, h *in vitro *stimulation of PBLs after pre-treatment of PBLs with rIL-2.

In PBL cultures there was no significant difference in IFN-γ gene expression between patient groups. There was a significant relationship between the type of exogenous stimulation and IFN-γ gene expression (*P *= 0.0001); there was a significant interaction between patient group and type of stimulation (*P *= 0.0002). Thus IFN-γ gene expression was lower in the PBLs of patients with sepsis in the presence of LPS, and IFN-γ gene expression was higher in PBLs of patients with sepsis in the presence of CD3ab (Figure 4).

In PBL cultures there was no significant difference in IL-7 gene expression between patient groups (Figure 4). However there was a significant relation between IL-7 gene expression and stimulus type, with IL-7 expression greater in the presence of LPS compared with control medium (*P *= 0.002). The interaction between patient Group and Stimulus type had a significant effect on IL-7 gene expression (*P *= 0.04), as in the presence of CD3ab, IL-7 gene expression was greater in PBLs from patients with sepsis than in control samples (*P *= 0.005).

When cultured PBLs of patients with sepsis and healthy controls were compared, there was no significant difference in IL-2 gene expression between patient Groups. There was a significant difference in IL-2 gene expression with Stimulus type (*P *= 0.002), with IL-2 gene expression greater in the presence of PMA compared with a control medium (*P *= 0.0002). The interaction between patient Group and type of Stimulus had no effect on IL-2 gene expression.

In parallel cultures in the presence of rIL-2, there was no significant difference in IFN-γ. IL-7, or IL-2 gene expression between Groups or with exogenous Stimuli (Figure 4).

There was no relation between Bcl-2 gene expression and patient Group or exogenous Stimulus. In parallel cultures in the presence of rIL-2, there was an interaction between patient Group and exogenous Stimulus which had a significant effect on Bcl-2 gene expression, (*P *= 0.0008) (Figure 4). On *post hoc *testing Bcl-2 gene expression was significantly lower in PBLs of patients with sepsis compared with controls, after culture in the presence of αCD3ab and rIL-2 (*P *= 0.005).

## Discussion

The onset of infection and subsequent occurrence of sepsis is linked with anomalous gene expression of γ_c _cytokines IL-2 and IL-7. IL-2 the prototypical member of the γ_c _cytokine family, is secreted after T cell receptor (TCR) stimulation [20] and acting as a T cell growth factor [21], plays a pivotal role in the T cell phenotype differentiation [22], contributing to the development of regulatory T cells and immune tolerance [23]. IL-2 produced by T cells in an autocrine manner in the initial stages of T cell activation, is essential for the generation and maintenance of effector and memory cells [24]. The actions of IL-2 are multi-faceted, as IL-2 has been shown to induce apoptosis of activated T cells [25]. It is plausible that the decrease in IL-2 gene expression which we observed in patients with gram negative infection and sepsis may reflect a failure of T cell activation.

Cell culture experiments indicated that treatment with exogenous IL-2 down regulated gene expression of the anti apoptotic marker Bcl-2 in activated T cells of septic patients. This effect of exogenous IL-2 may represent activation induced leukocyte apoptosis, which is a well described effect of IL-2 [25]. Furthermore, IL-2 attenuated inducible IFN-γ gene expression, which effect is plausibly related to the pro apoptotic effects of IL-2 in activated T cells. These data would preclude consideration of IL-2 as a sepsis immune adjuvant, and contributes to the increasing recognition that apoptotic processes have a salient role in septic shock pathophysiology [26,27].

IL-7 is a recognised lymphocyte survival factor [28]. Produced by stromal and epithelial cells in the bone marrow, by the thymus and by fibroblastic reticular cells in T zones of secondary lymphoid organs, and by dendritic cells, IL-7 regulates T cell activation by dendritic cells [29,30]. The bioavailability of IL-7 is determined both by its production and consumption by CD4^+ ^T cells [2]. Thus lymphopenia in humans should be associated with reciprocal increases in IL-7 levels [31]. In this study lymphopenic patients with severe sepsis had decreased IL-7 gene expression, compared to controls and patients with infection. Direct T cell stimulation increased IL-7 gene expression of PBLs from septic patients rather than control subjects, suggesting that IL-7 production by peripheral lymphocytes requires prior lymphocyte activation. Excess IL-2 abolished this difference in IL-7 gene expression, which may also represent a side effect of activation apoptosis. While IL-7 may have the potential to reverse sepsis induced apoptosis and rhIL-2 related apoptosis in PBL of septic patients, this will require further investigation and is beyond the scope of the current project.

IL-7 negatively regulates cell surface expression of its cognate receptor CD-127 [32]. This receptor, CD-127, is expressed on naive and memory T cells, but not on effector T cells. In this study, patients with sepsis exhibited a marked reduction in CD127^-_^ve T cells, which includes the subpopulation of effector T cells, and suggests that the characteristic lymphopenia of sepsis primarily involves effector T cells. IL-7 regulates T cell homeostasis in part by increasing the expression of Bcl-2, and by inhibiting the expression of the pro-apoptotic factors Bax and Bim [33,34]. While we observed differential gene expressionof Bcl-2 and Bim in patients with sepsis, these effects were not obvious immediately after surgery in the initial stages of infection.

In this study of adults with bacterial infection, IL-15 gene expression was similar in all patient groups. This is not surprising as IL-15, in concert with IL-7, acts as a regulator of CD8+ T cells, whose primary role is in the immune response to viral infection, and not bacterial infection [35]. Using a well-established qRT-PCR protocol we could not find any detectable IL-21 mRNA in PBL's of humans with sepsis, suggesting that IL-21 is predominantly produced by lymphocytes populations other than PBL's and is not of pivotal importance in the immune response to infection in adults. This is consistent with existing literature which implicates IL-21 in the pathogenesis of atoimmune diseases such SLE, diabetes and psoriasis [36,37].

IFN-γ produced by Th1 CD4 cells and antigen presenting cells (APCs) augments phagocytic bactericidal activity in patients with infection [38,39], and is a useful metric of host immune response to infection, and we considered it as such in the cell culture experiments. In this study down-regulation of IFN-γ gene expression was evident in cultured PBLs of septic patients. Furthermore, the reported attenuation of IFN-γ gene expression in cultured PBLs of patients with sepsis was corrected by direct T cell stimulation, but was refractory to stimulation with monoctye specific ligands. Thus, it seems plausible that the observed down-regulation of IFN-γ gene expression in sepsis is indicative of defective antigen presentation by APCs such as monocytes, rather than an intrinsic T cell defect. It is notable that our attempt to circumvent this defect in APC dysfunction using rIL-2 in cell cultures failed, as this intervention appeared to augment pro apoptotic signals and inhibited inducible IFN-γ and IL-7 production.

The link between sepsis and lymphocyte apoptosis is well established, with several investigators reporting down-regulation of gene expression of the anti apoptotic protein Bcl-2 and upregulation of gene expression of antagonistic pro-apoptotic proteins, such as Bid and Bax. In addition, there is evidence from animal sepsis models that manipulation of these regulators of apoptosis may improve outcome [40-42]. However, we noted that Bcl-2 and related genes were also dysregulated in patients with infection, but to a lesser extent than in septic patients. Furthermore, prior to the onset of infection we observed changes in IL-2 and IL-7 gene expression without any change in expression of the Bcl-2 related genes. As the effects of IL-2 and IL-7 are mediated by regulating Bcl-2 and related genes, it is plausible that the reported changes in Bcl-2 related genes in septic patients reflect underlying changes in gene expression of the gamma chain family of cytokines, specifically IL-2 and IL-7.

This study elaborates upon our existing knowledge of the immune suppression which characterises sepsis. While the severity of organ failure in septic patients can be directly related to humeral factors, such as IL-6 and complement [43,44], the concomitant immune suppression can be linked to dysregulation of the immune response by PBL. Thus, in patients with sepsis, and who fail to recover from sepsis, PBLs produce less of the effector cytokines that enhance phagocytic bactericidal activity such as IFN-γ and TNFα. Furthermore, PBLs of septic patients exhibit anaomolus production of cytokines mediating T cell activation [8]. In addition, surface expression of MHC class II molecules is down-regulated in monocytes of septic patients and septic patients who encounter nosocomial infections [45]. Lastly, gene expression of the cytokines required to maintain T cell homeostasis appears to be deficient in the PBL of septic patients. Hence, in septic patients there appears to be a complex multifactorial immune paresis. The complexity of this immune paresis suggests that immune adjuvant sepsis therapies may need to be multimodal and provided on an individualised basis.

## Conclusions

In conclusion there is a relation between aberrant gene expression of the γc family of cytokines, and the human immune response to infection and the onset of sepsis.

## Key messages

• The occurrence of infection and sepsis are linked with deficient IL-2 and IL-7 gene expression.

• Similar patterns of cytokine expression are observed both in sepsis and prior to the onset of infection.

• Onset of infection is associated with deficiency of cytokines that regulate T cell homeostasis.

• Changes in gene expression appear to precede the onset of clinical infection.

## Abbreviations

γc: Gamma chain cytokines; ABI: Applied Biosystems; APC: antigen presenting cells; cDNA: complementary deoxyribonucleic acid; Ct: crossing threshold; DNA: deoxyribonucleic acid; DTT: dithiothreitol; EDTA: ethylenediaminetetraacetic acid; ELISA: Enzyme Linked Immunosorbant Assay; FCS: fetal calf serum; HEPES: N-2-Hydroxyethylpiperazine-N'-2-Ethanesulfonic Acid; ICU: intensive care unit; IFN-γ: interferon-gamma; IL: interleukin; LPS: lipopolysaccharide; mAB: monoclonal antibody; mRNA: messenger ribonucleic acid; NK: natural killer; PBLs: peripheral blood leukocytes; PFA: paraformaldehyde; PMA: phorbol myristate acetate; POP: post-operative pneumonia; rIL-2: recombinant interleukin-2; RNA: ribonucleic acid; RT-PCR: real time polymerase chain reaction; SAPS II: Simplified Acute Physiology Score II; SOFA: Sequential Organ Failure Assessment; TCR: T cell receptor; Treg: regulatory T cells.

## Competing interests

Dr. M White, Dr. T Ryan, Professor D Kelleher and Dr. R McManus have a patent application pending on the relation of cytokine mRNA levels to the risk of developing nosocomial pneumonia after thoracic surgery. Dr. T Ryan and Dr. R McManus are co-supervisors of a project funded by the Irish Health Research Board into gene expression patterns in infection and sepsis.

## Authors' contributions

MW, TR and RM contributed to the conception and design of this study, acquisition of data, analysis and interpretation of data and manuscript preparation. VM and RG contributed to acquisition and interpretation of data and manuscript preparation. DD and DK contributed to the conception and design of this study and manuscript preparation. All authors have read and approved of the final manuscript.
